# Untangling the tangled relationship between cognitive and psychological comorbidities in epilepsy: Bidirectionality and mediation

**DOI:** 10.1111/epi.18589

**Published:** 2025-07-31

**Authors:** Bruce P. Hermann, Aaron F. Struck, Qirui Zhang, Sam S. Javidi, Joseph I. Tracy

**Affiliations:** ^1^ University of Wisconsin Madison Wisconsin USA; ^2^ Department of Neurology, Farber Institute for Neuroscience Thomas Jefferson University Philadelphia Pennsylvania USA

**Keywords:** cognitive impairment, epilepsy, functional reserve, multimorbidity, psychopathology

## Abstract

**Objective:**

Cognitive and psychological abnormalities are known to be frequently occurring complications of the epilepsies, but their patterns of co‐occurrence, directions of effect, underlying mechanism(s), and causal pathways remain uncertain. The intent of this investigation was to advance understanding of these issues in patients with temporal lobe epilepsy (TLE).

**Methods:**

A total of 121 patients with TLE were administered a comprehensive neuropsychological battery, and symptoms of diverse psychological syndromes were assessed with a standardized psychological inventory. The cognitive and psychological data were reduced by factor analysis (FA) to broad‐ and narrowband indicators that were then examined by partial least squares structural equation modeling (PLS‐SEM) to inform the presence, strength, direction, and mediators of cognitive–psychological relationships.

**Results:**

FA identified overarching metrics of cognition (g) and psychopathology (p) as well as their correlation and underlying factors wherein g contained three indicators (language/memory, perceptual/nonverbal reasoning, information/processing speed) and p contained two indicators (externalizing, internalizing). PLS‐SEM demonstrated direct, bidirectional associations between internalizing psychopathology and language/memory, indicating these factors express a strong interdependency. Mediation modeling showed an index of functional brain reserve served to explain this selective, shared influence, as well the relationship between the broader concepts of p and g, indicating origin in a common psychological/cognitive mechanism.

**Significance:**

Neuropsychological and psychological comorbidities of TLE are represented by both broadband and narrowband indicators of clinical and theoretical relevance. These indicators exhibit multidimensionality, multimorbidity, and bidirectionality that point to a shared neurobiological core best expressed and explained by the mediating influence of brain functional reserve.

**Key points**

- Neurocognitive and psychological comorbidities in TLE suggest a shared neurobiological substrate mediated by brain functional reserve.
- The findings point to the intertwined nature of cognitive and psychological functioning in TLE.
- Such comorbidities indicate that the presence of dysfunction in one domain places individuals at risk for dysfunction in the other.

## 1 INTRODUCTION

Abnormalities in cognition and behavior, often referred to as neurobehavioral comorbidities,^1^ are well recognized complications of the child and adult epilepsies that can not only adversely impact the quality of life of affected patients but also alter their life trajectories in unfortunate ways.^2^, ^3^ Several meaningful trends characterize the extensive research literature on these important issues.

First, from a historical perspective, cognitive and psychological abnormalities were examined in independent streams of research—each separately interrogated in a number of ways. These efforts included characterization of their overall prevalence, or the prevalence of specific cognitive (e.g., memory)^4^ or psychological/behavioral (e.g., depression) abnormalities,^5^ their association with diverse clinical epilepsy factors (e.g., age at seizure onset, seizure frequency/severity, epilepsy syndrome), and lastly, the course or trajectory of these separate abnormalities over time.^6^, ^7^ These investigations resulted in a formidable amount of information published over decades of empirical investigation.

Second, a major conceptual shift occurred upon awareness that cognitive^8^ and behavioral^9^ complications could be present at, or even antecedent to, the onset and diagnosis of epilepsy, including among drug‐naïve patients. Although there is ample evidence consistent with the longstanding assumption that these comorbidities can develop and potentially worsen after disease onset and diagnosis,^10^, ^11^, ^12^ there is now a clearer picture of the directional nature of comorbidity–epilepsy relationships.^13^, ^14^

Third, a more recent development has been the appreciation that cognitive and behavioral abnormalities may co‐occur, resulting in so‐called “multimorbidity,” contributing substantially to the burden epilepsy patients must bear. Furthermore, the notion of multimorbidity can be extended to include the presence of more than one cognitive (e.g., memory) or psychological/behavioral (e.g., depression) abnormality, thereby capturing the variety of cognitive (e.g., memory + language + executive deficits)^15^ or psychological (e.g., depression + anxiety)^16^ problems that may exist. These patterns of multimorbidity could be attributable to shared disease‐related risk factors (e.g., epilepsy severity), shared neurobiological abnormalities (e.g., different expressions of a common brain network or pathophysiology), shared polygenetic background, or shared social determinants of health. Most importantly, the interrelationships and interdependencies among these many dimensions of burden and abnormality need to be clarified and modeled to advance understanding of multimorbidity and its impact on epilepsy.

Fourth, a general observation is that the research to date has been notably “model‐agnostic.” That is, longstanding conceptual and empirical models of human cognition, such as the Cattell–Horn–Carroll approach,^17^ as well as conceptual and empirically based approaches to psychopathology, such as the Research Domain Criteria^18^ or the Hierarchical Taxonomy of Psychopathology,^19^ have been surprisingly absent in epilepsy research despite the utility of these approaches in other fields. Because the focus in epilepsy has been largely on “fine grain” cognitive and psychological/behavioral issues and related approaches, potentially useful overarching metrics, including g for cognition and p for psychopathology, as well as their underlying factor structures that have been demonstrated to be relevant, useful, and predictive in other fields, have been underinvestigated in epilepsy, with their utility remaining to be examined.

Here, we attempt to break new ground through the use of factor analytically derived metrics of cognitive and psychological status, interrogating their interrelationships to test for dependencies that might inform their shared neurobiological substrate(s), potentially yielding new behavioral phenotypes to explain their co‐occurring effects in epilepsy. Specifically, we test whether a patient's status in one comorbidity (cognitive or psychological/behavioral) drives and predicts status in the other. The overarching g and p latent factors (LFs) and others are examined at several dimensional levels (i.e., decomposing the cognitive and psychological/behavioral domains into one versus multiple LFs). Given the substantial literature pointing to the effects of psychiatric conditions on neurocognitive status and integrity (e.g., among patients with schizophrenia,^20^ anxiety,^21^ bipolar disorder,^22^ and depression^23^, ^24^, ^25^), we predict that the latent dimensions underlying the psychological/behavioral measures will be strong drivers of latent dimensions of neurocognition, but we also interrogate for bidirectionality. In the setting of temporal lobe epilepsy (TLE), we first model the potential interdependent relationships between these cognitive and psychological comorbidities. Next, in an attempt to identify a functional construct that might help explain the comorbidity, we examine the role played by a measure of functional brain reserve as a potentially mediating influence in the relationship between these cognitive and psychological domains.

## 2 MATERIALS AND METHODS

### 2.1 Participants

All eligible patients with drug‐resistant focal TLE (*n* = 121; 73 left‐sided, 48 right‐sided) were included from the Thomas Jefferson University Comprehensive Epilepsy Center (TJUCEC), with completed neurocognitive/psychological assessment as part of their presurgical evaluation. All patients were candidates approved for thermal ablation or resection of the ictal temporal lobe (details provided in Appendix S1). The temporal lobe seizure focus was determined by multimodal data (surface video‐electroencephalography, magnetic resonance imaging, positron emission tomography, neuropsychological assessment) and a consensus decision of the TJUCEC surgical conference committee.^26^, ^27^ The TLE sample demographics, clinical characteristics, and mean values on Personality Assessment Inventory (PAI) subtests and cognitive measures are provided in Table 1.

**TABLE 1:** Sample demographic and clinical characteristics and mean cognitive and personality assessment inventory scores.

TLE subjects, *N* = 121	Value	g (cognitive measure)	Score type	Score	p (PAI subscale)	Score type	Score
Age, years	41.1 ± 13.8	Vocabulary	SS	9.4 ± 2.8	Somatic complaints	TS	61.2 ± 11.0
Sex, male/female	62/59	Similarities	SS	9.4 ± 2.6	Anxiety	TS	53.4 ± 9.9
Years of education	14.4 ± 2.3	Semantic fluency	SS	7.7 ± 3.1	Anxiety‐related disorders	TS	50.7 ± 10.0
TLE laterality, R/L	73/48	Letter fluency	SS	7.7 ± 3.1	Depression	TS	55.0 ± 10.6
Age at epilepsy onset, years	24.4 ± 15.0	BNT	TS	39.1 ± 11.3	Borderline features	TS	50.6 ± 10.4
Duration of epilepsy, years	16.7 ± 16.1	Matrix reasoning	SS	10.3 ± 3.1	Paranoia	TS	50.4 ± 9.6
		Digit span	SS	9.1 ± 2.9	Schizophrenia	TS	52.0 ± 10.8
		WCST PR	TS	47.0 ± 11.3	Mania	TS	47.2 ± 9.5
		WCST CC	RS	4.7 ± 1.9	Antisocial features	TS	48.8 ± 9.5
		TMT B	TS	45.3 ± 12.2	Alcohol features	TS	47.5 ± 7.4
		TMT A	TS	44.9 ± 13.5	Drug problems	TS	50 ± 8.6
		Coding	SS	8.8 ± 2.5			
		Logical memory 1	SS	9.0 ± 3.2			
		Logical memory 2	SS	8.2 ± 3.5			
		CVLT TL	TS	47.9 ± 12.0			
		CVLT TLDFR	ZS	−.5 ± 1.3			
		Block design	SS	8.7 ± 2.7			
		ROCF copy	RS	29.2 ± 6.1			
		Pegboard R	TS	38.6 ± 11.2			
		Pegboard L	TS	38.6 ± 11.2			

*Note*: Continuous variables are presented as mean ± SD.

Abbreviations: L, left; PAI, Personality Assessment Inventory; R, right; RS, raw score; SS, scaled score, mean of 10, SD of 3; TLE, temporal lobe epilepsy; TS, *T*‐score, mean of 50, SD of 10; ZS, *Z*‐score, mean of 0, SD of 1. Wechsler Adult Intelligence Scale, version III or IV subtests: Vocabulary, Similarities, Matrix Reasoning, Digit Span, Digit Symbol Coding, Block Design. Wechsler Memory Scale version III or IV: Logical Memory I (1) or II (2). Trail Making Test, B (TMT‐B), A (TMT‐A). Boston Naming Test (BNT). Wisconsin Card Sort: Perseverative Responses (PR), Categories Completed (CC). California Verbal Learning Test, version II or III: Total Learning (TL), Long Delay Free Recall (LDFR). Controlled Oral Word Association Test: Letter fluency, Semantic fluency.

### 2.2 Cognitive and behavioral measures

The psychological data included the 11 clinical scales from the PAI,^28^ which included scales assessing somatic complaints, anxiety, anxiety‐related disorders, depression, mania, paranoia, schizophrenia, borderline features, antisocial features, alcohol problems, and drug problems (see Table S1 for description of subscales). The cognitive data included 20 tests representing the traditional cognitive domains of language, perceptual/constructional, verbal and visual memory, executive function, and speeded abilities. Age‐ and gender‐normalized (when available) test scores were used for factor analysis (FA) of the cognitive data.

### 2.3 Statistical analyses

#### 2.3.1 Factor analysis

The psychological/behavioral and cognitive data were subjected to exploratory FA. Both sets of tests were subjected to the Kaiser–Meyer–Olkin test^29^ to assess sample measure adequacy for FA. See Appendix S1 for details.

#### 2.3.2 Partial least squares structural equation modeling

**FIGURE 1:**
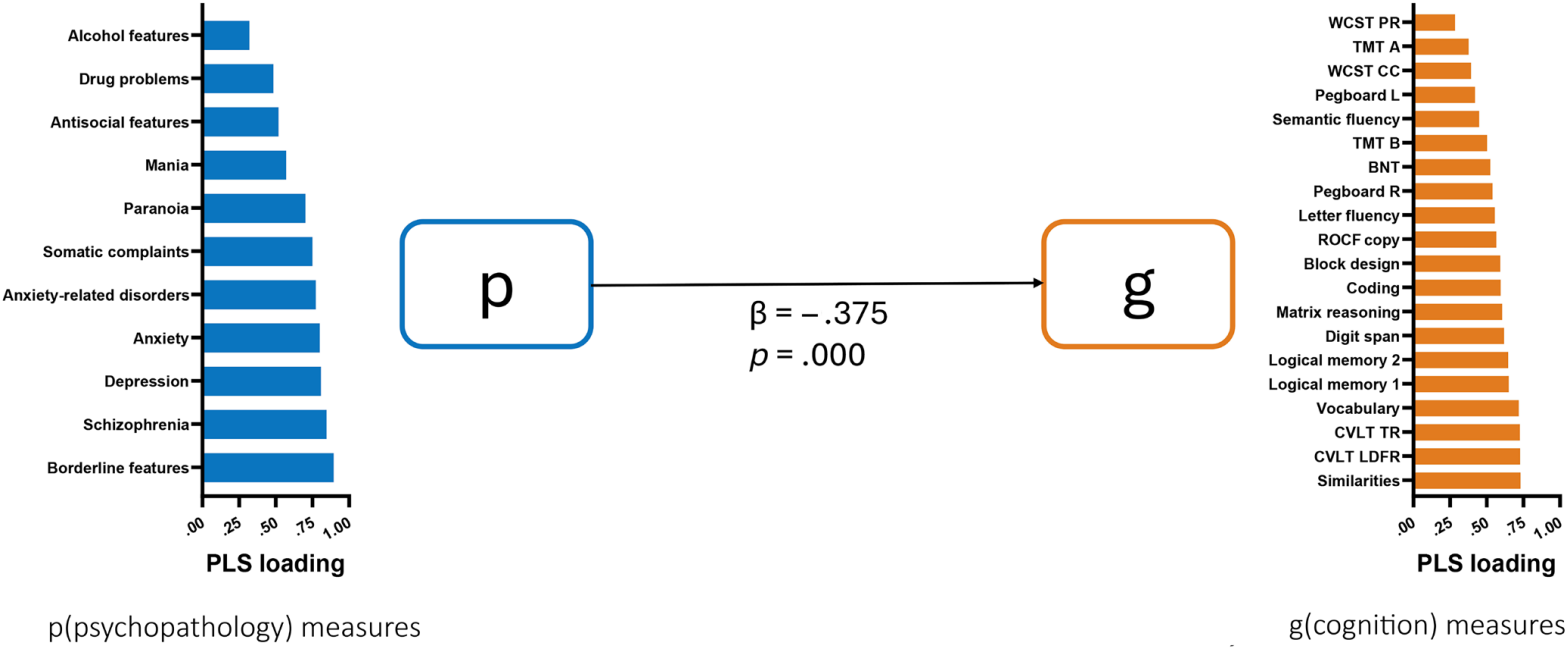
Low‐dimensional partial least squares (PLS) structural equation modeling model (model 1), with one p and one g latent factor. Bar graph shows indicator loadings for each latent factor. Direct effect coefficient and *p*‐value are shown. Psychopathology measures from PAI (p). Cognition measures (g, see Table 1 legend for test name and associated abbreviation).

**FIGURE 2:**
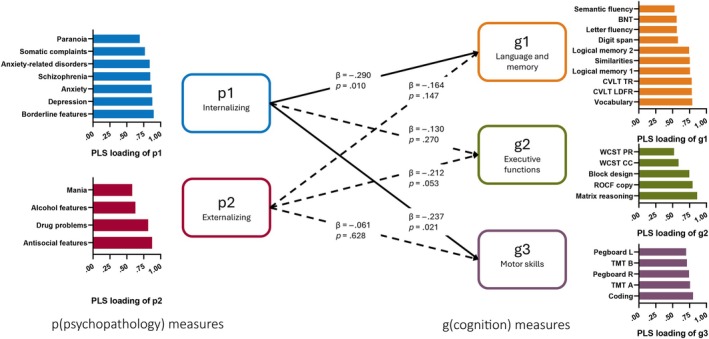
High‐dimensional partial least squares (PLS) structural equation modeling model (model 2), with two p and three g latent factors. Bar graph shows indicator loadings for each latent factor. Direct effect coefficients and *p*‐values are shown. Psychopathology measures from PAI (p). Cognition measures (g, see Table 1 legend for test name and associated abbreviation).

Partial least squares structural equation modeling (PLS‐SEM; https://www.smartpls.com)^30^ was performed to determine whether the latent cognitive factor(s) yielded by the FA showed a dependency on the psychological measures as captured through their FA‐based LF(s). PLS‐SEM provided feature reduction followed by regression analyses of the underlying latent dimensions to model and quantify relationships between multiple independent (11 psychological measures, i.e., PAI subscales) and dependent variables (18 cognitive measures), emphasizing direct effects on the dependent variable models (groups permutation, 1000 bootstrapped samples). The measured psychological and neuropsychological measures are referred to in this context as indicators. Each set of indicators were decomposed to form one or more LFs. In model 1, only one LF was modeled for each of the indicator sets (p, g; see Figure 1). In model 2, we increased the dimensionality of our LFs. To construct this new dimensionality, we utilized as indicators in the decomposition the variables that weighted heavily on each of the factors in the two‐factor psychological FA and three‐factor cognitive FA solution (see Figure 2). More specifically, sets of seven and four psychological variables were decomposed into their respective LFs, yielding two psychological LFs (p1 and p2). Similarly, sets of 10, five, and five neuropsychological variables were decomposed into their respective LFs, yielding three cognitive LFs (g1, g2, and g3). Each of the PLS‐SEM models tested for the direct influence of the psychological LF(s) on the cognitive LF(s), with each model yielding weighted coefficients (β) quantifying the direct effect of each psychological LF (p) on each cognitive LF (g). To model the potential influence of premorbid functional reserve on the observed relationships between the latent structures of our psychological and cognitive measures, we reconstructed our high and low PLS‐SEM models with a measure of functional reserve inserted as a mediator of the relationship between our p and g LFs. As we did not want to presume the relationship between psychological and cognitive status was unidirectional, we reran model 2 reversing the direction of influence and prediction (i.e., LFs of g → LFs of p).

## 3 RESULTS

Demographic and clinical history characteristics and mean values of the cognitive and psychological measures are shown in Table 1.

### 3.1 Factor analysis

FA on the PAI subscales resulted in an overall measure of sampling adequacy (MSA) of .88, with all factors > .6. Minimum average partial test (MAP), by 1976 and 2000 criteria, suggested two reliable factors confirmed by scree plot (see Figure S1A,B and Table S2 for relevant FA statistics). Interpretively, the FA on the PAI subscales suggested a largely internalizing and inward‐directed psychopathology for the first factor, whereas the second factor was reflective of more externalizing and outward‐directed psychopathology. Such factors have been reliably reported in the psychopathology literature.^31^ FA on the cognitive data yield at MSA of .8, with all factors > .6. MAP suggested seven factors, but scree plot suggested three (see Figure S2A,B and Table S2 for relevant FA statistics). The three‐factor solution aligned well with current understanding of associations among cognitive tests and underlying latent cognitive concepts. The three factors were characterized as language and memory, perceptual/nonverbal reasoning (P/NVR), and information/motor processing speed (I/MPS).

### 3.2 PLS‐SEM models

#### 3.2.1 Low‐dimensional p/g model (PLS‐SEM model 1, i.e., one psychological, one cognitive LF)

The findings indicated that the cognitive LF has a reliable dependency on the psychological LF (model‐adjusted *R* ^2^ = .153; see Figure 1). The direction of the dependency indicated that higher levels of cognitive performance depended on higher levels of psychological health (i.e., less psychopathology). The bar chart in Figure 1 shows the PLS‐SEM loadings for the indicator (observed) variables on their respective psychological and cognitive LFs.

#### 3.2.2 High‐dimensional p/g model (PLS‐SEM model 2, i.e., two psychological and three cognitive LFs)

The results revealed two of the three cognitive LFs (language/memory [L/M], I/MPS) showed a reliable dependency on one of the psychological LFs (internalizing; L/M, adjusted *R* ^2^ = .135, *p* = .021; I/MPS, adjusted *R* ^2^ = .056; see Figure 2). The relationship between the P/NVR LF and the externalizing LF indicated a tendency for the former to depend on the latter (i.e., statistical trend, *p* = .053). The bar chart in Figure 2 shows the PLS‐SEM loadings for the indicator (observed) variables on their respective psychological and cognitive LFs.

#### 3.2.3 Cross‐correlation

**FIGURE 3:**
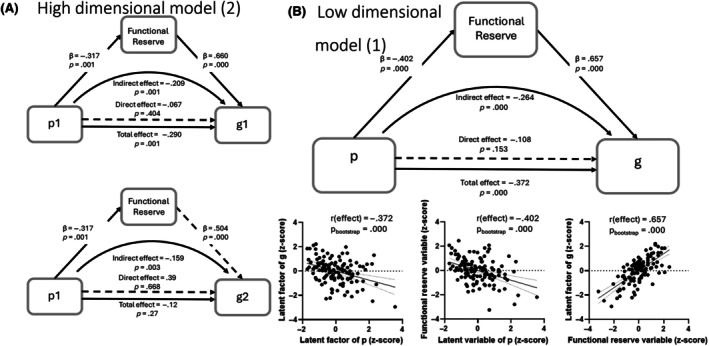
Partial least squares structural equation modeling mediation models involving functional reserve. Full models were run but only latent factors with significant mediation effects are shown here. (A) Mediation of high‐dimensional model. (B) Mediation of low‐dimensional model. Direct and indirect coefficients are shown with *p*‐values.

Figure S3A displays the cross‐correlation between the observed psychological and cognitive measures. Figure 3B displays the correlation between the LFs of the low‐dimensional model (also referred to as PLS‐SEM model 1) involving one independent variable (p) and one dependent variable (g). The cross‐correlations of the LFs for the high‐dimensional p and g model (PLS‐SEM model 2) are shown in Figure S4.

#### 3.2.4 Bidirectional effects

To test for reversed or bidirectional effects among the LFs of the high‐dimensional model, we reran the PLS‐SEM testing for the influence of the cognitive LFs on the psychological LFs. The results reproduced one of the original p → g associations, indicating that the relationship between the internalizing LF and the L/M LF was bidirectional (adjusted internalizing *R* ^2^ = .124, *p* = .006; see Figure S5). The direction of this interdependency indicated that higher levels of cognitive performance depended on higher levels of psychological health (i.e., less psychopathology).

#### 3.2.5 Mediation modeling

Functional reserve was indexed by a measure of crystallized intelligence (Wide Range Achievement Test; see Stern et al.^32^, ^33^ for a discussion of reading decoding as an index of crystallized intelligence and, more broadly, as an index of functional reserve). The results of this mediated PLS‐SEM model revealed a loss of all direct effects between our two psychological and three cognitive LFs (functional reserve, adjusted *R* ^2^ = .148; see Figure 3A). Two indirect effects emerged, both indicating that functional reserve mediated the relationship between the internalizing psychological LF and two of the cognitive LFs. Namely, with the mediation of functional reserve, the internalizing LF displayed an indirect effect on both the L/M (indirect effect = −.209, *p* = .001; direct effect = −.067, *p* = .404) and P/NVR LFs (indirect effect = −.159, *p* = .003; direct effect = .39, *p* = .668). We reran this mediated PLS‐SEM model with the direction of mediated influence reversed (i.e., testing the dependency of the internalizing LF on the L/M and P/NVR cognitive LFs). The same results held, indicating that the mediating influence of functional reserve on the relationship between the internalizing LF and L/M and P/NVR cognitive LFs was bidirectional.

We also tested model 1 for mediation by functional reserve. The results indicated a loss of the direct relationship between p and g, and the presence of functional reserved as a significant mediator (adjusted *R* ^2^ = .155, indirect effect = −.264, *p* = .000; direct effect = −.108, *p* = .153; see Figure 3B). As with our main unmediated PLS‐SEM models, the direction of the influence was as expected, indicating that higher psychopathology influenced lower reserve, which in turn yielded lower cognitive status.

Our PLS‐SEM methodology allowed us to establish reliable and valid measurement models for the p and g constructs and revealed the presence of a reliable mediator for relating the broad constructs of p and g, as well for modeling the relationship between select higher dimensional constructs embedded within p and g.

## 4 DISCUSSION

Several noteworthy aspects follow from this investigation, including the following: (1) identification of single overarching metrics of psychopathology (p) and cognition (g) in patients with TLE, demonstrating that the nature of their relationship informs the core issue of multimorbidity in epilepsy; (2) clarification of the higher dimensional factors underlying the broad constructs of both p and g; (3) mapping of the higher dimensional associations between the indicators of p and g, showing that key, mediated, bidirectional associations exist; and (4) demonstration of the relevance of these findings to the underlying neurobiology of TLE by providing clear evidence of a mediated link between the psychopathologic and cognitive presentations of the disorder. Each of these points will be addressed in turn below.

### 4.1 Overarching metrics of psychopathology (p) and cognition (g) in TLE

The clinical neuropsychology of epilepsy, and particularly the neuropsychology of TLE, have been characterized by a focus on specific metrics of discrete cognitive abilities (e.g., memory, language, executive function), and 20 such metrics were available for our analyses. The implicit unstated assumption in most clinical and research use is that these diverse metrics are specific reflections of ability. A similar philosophy is evident in the realm of psychopathology, where the weight of focus tends to be on specific disorders (e.g., depression, anxiety). But considerable evidence exists, overwhelmingly but not totally from outside epilepsy research, that points to the significant collinearity that exists across measures of cognition (i.e., the positive manifold), which is also true for measures of psychopathology.

Furthermore, these indicators can be subsumed under an even broader umbrella, including g for cognition and p for psychopathology. Our factor analyses demonstrated this collinearity well by providing robust evidence for a general factor underlying the observed measures of both domains. There has been only a minimal amount of effort directed to g and especially p in epilepsy, and with few exceptions^34^, ^35^ and even less consideration to the conceptual or theoretical models of these broad constructs that are of such import in other fields. A starting point given by our data is to show that both g and p can be identified in traditional psychological and cognitive test batteries in TLE.

Most directly relevant to the issue of multimorbidity in epilepsy is our demonstration of a significant association between p and g that also informs the broader mutual dependence of these major cognitive and behavioral comorbidities. Such mutual dependence points to a potential shared neurobiological core, explanatory to both the p and g constructs. We argue that our data on mediation, to be discussed below, points to a behavioral phenotypic core expressed through our functional reserve measure. We will refer to this phenotypic core as “p/fr/g.”

### 4.2 Indicators of p and g

Although a significant relationship between g and p is informative and important, also of interest is the higher dimensionality underlying the matrix of specific indicators quantifying p and g. Here, we found that g was composed of three specific factors, including L/M, P/NVR, and I/MPS. Although specific cognitive metrics may be of primary clinical interest, memory for example, verbal memory is found here to be embedded in a factor along with several other abilities dominated by, but not limited to, language‐dependent skills. Similarly, the 11 psychological measures were composed of two LFs, capturing the classic internalizing and externalizing behavioral dimensions reported for decades in the personality and psychiatric literatures.^36^ Yet, within each of these latent dimensions, a diverse set of behavioral/psychopathologic symptoms are subsumed. The covariation in these cognitive or psychological subcomponents is rarely considered in the literature, with one of the components “covaried” out to more purely examine a specific influence in the setting of an experimental design. So at a fundamental level, in the realm of cognition, “memory problems” cotravel with other cognitive issues (language abilities) representing multimorbidity within cognition. Regarding psychological symptoms, “depression” cotravels with a number of other behavioral problems (anxiety, somatic complaints, paranoia, borderline features), representing multimorbidity within psychopathology. So multimorbidity can be appreciated at a very broad level (p with g) as well as at narrower levels (n.b., within indicators of cognition and within indicators of psychopathology).

### 4.3 Associations across p and g factors and their implications for understanding directional influences of multimorbidity

Given the multimorbidity that may be evident in patients with epilepsy, increasing attention has been directed to their association or influence on one another. For instance, in a preoperative cognitive assessment, does depression impact the overall cognitive profile or impact important cognitive domains in particular (such as memory), and if so, how strong is this relationship? In examining the dependence of g‐factors on p‐factors (Figure 2), we found that the internalizing dimension of p exerted significant direct effects on the learning/memory and I/MPS factors of g, with P/NVR showing no apparent influence from this internalizing factor. Furthermore, there was no clear and strong direct dependence of any of the g‐factors on the externalizing dimension of p (n.b., the dependence of the P/NVR factor was a statistical trend).

Importantly, certain of these associations appeared to be a “two‐way street.” When the direction of influence between the p and g LFs was reversed, there was only a dependence of internalizing p‐factor on the L/M factor of g. The bidirectionality of this effect indicated that lower levels of internalizing psychopathology (or vice versa, lower levels of L/M) reduced neurocognitive status (or vice versa, psychopathology status). An important observation is that the inner mental processes inherent to the psychopathology of the internalizing factor are likely verbal in nature. Similarly, the cognitive skills that loaded on the L/M factor generally involve internal verbal activity (e.g., deep semantic encoding, lexical search) as part of their processing stream. Thus, it is intuitive to find a bidirectional relationship between these inherently verbal processes. Our data highlighted that there are robust interdependencies among specific, finer grained dimensions of p and g.

### 4.4 Mediated association between p and g and implication for understanding their shared neurobiological substrate

In both our lower and higher dimensional PLS‐SEM models, we discovered an indirect‐only form of mediation, suggesting that the presence of an outside factor accounts for all of the observed direct relationships between the LFs, indicating that only in the setting of functional reserve was the theoretical relationship between g and p complete. With both lower and higher dimensionality, our data showed that as a best‐case scenario there must be at least one other construct brought into the theoretical framework for the g and p relationship to be empirically valid. Here, we obtained evidence that the construct of functional reserve is that reliable mediator. However, at higher dimensionality not all of the subconstructs embedded within the broad p and g factors were mediated (n.b., internalizing psychopathology had a mediated influence on L/M and P/NVR, not I/MPS).

Accordingly, our data pointed to a superseding mediated relationship between the two forms of internal processing described above. That is, the most reliable relationship between the internalizing psychological factor and the forms of verbal processing noted above (verbal fluency and speech, expressive vocabulary, and verbal auditory short‐term memory) go through and rely upon the level of functional brain reserve. Similarly, the relationship between the internalizing and the P/NVR LFs was mediated. The direction of these relationships also appeared intuitive, as it indicated that the dedication of energy and resources to an internal mental state would reduce functional reserve with regard to the production of perceptual (i.e., externally oriented) nonverbal problem‐solving strategies.

Allowing for the mediation of functional reserve in the causal chain between our higher dimensional p and g LFs brings to the surface the full set of influences on multimorbidities. Thus, our mediated models showed that the internalizing p factor influences both the broad and narrower dimensions of g, but only indirectly. Accordingly, our mediation data allowed us to adjust our previous consideration of the p and g constructs to consider a more complex cause–effect relationship, making clear that they share a common basis in the overall health status of the brain (i.e., functional reserve), raising the possibility this shared basis is also expressed at a neurobiological level.

## 5 CONCLUSIONS

The presented factor analytic and PLS‐SEM methods allowed us to establish reliable and valid measurement models for the p and g constructs, both for broad conceptions of p and g, and to select higher dimensional constructs embedded within them. We conclude there are both broad and specific multimorbidities at work, with some of the latter bidirectional such that the dependency goes both ways. Importantly, the data demonstrated that an index of functional reserve is an appropriate mechanism to explain the interdependency of the psychological and cognitive constructs. Our modeling demonstrated that the broad constructs of p and g share 37.2% of their variance when accounting for the mediation of functional reserve compared to only 15.3% without such mediation. With this mediated model, we have identified a potential underlying mechanism that may govern the relationship between the p and g dimensions and, crucially, be determinative of the psychological and cognitive clinical presentations of TLE. As the concept of functional reserve has strong a priori theoretical and conceptual support in the literature on brain reserve,^37^, ^38^, ^39^ we argue that it measures an important brain capacity that is determinative of the relationship between both a broad and a more select set of g and p features as captured by our internalizing, L/M, and P/NVR LFs.

The concept of functional reserve does not simply argue for a transdiagnostic relation between psychopathology and cognition, which has been discussed in the literature,^40^, ^41^, ^42^ but points to the mediating brain state that may be at the neurobiological core of the p/g relationship. Functional reserve may be a surrogate representative of several constructs, either alone or in combination, reflecting (1) early brain health and/or genetics; (2) the social determinants of psychological processes and behavior, including cognition; and (3) the potential for developing problem‐solving and coping skills that are critical to the formation of personality styles and cognitive abilities. We acknowledge that there are likely other systematic influences on psychological and cognitive status and their mutual dependence (e.g., socioeconomic burden, stress coping capacity, genetics). Clearly, further work needs to be done to fully delineate the potential cause–effect relationships we have described here, as the nature of mediation effects may change related to epilepsy clinical factors and normal aging. For instance, it will be important to test whether these psychological/cognitive relationships differ in newly diagnosed versus chronic epilepsy, or in other epilepsy subgroups.

To further delineate the neurobiological basis of the mutual dependence and the mediated mechanism we report for p and g, we plan to utilize structural and functional imaging data to test whether the common “neuro [g] psycho [p] logical” behavioral phenotype we described here in terms of reserve is expressed at a functional and structural brain systems level, pursued via imaging modalities. We believe our results highlight the importance of accounting for general psychological factors and functional reserve when explaining the determinants of g‐factors, and vice versa. We hope we have achieved our goal of demonstrating the importance of representing broad latent dimensions of psychological and cognitive status, and potential mediating influences (p/fr/g), when constructing neurobiological models to explain behavioral comorbidities in neurological disorders such as TLE. Lastly, regarding clinical implications, by showing a linkage and mediation between these comorbidities, our findings will facilitate targeted interventions that simultaneously address both the psychological and cognitive symptoms of TLE, thereby enabling early, efficient individualized treatment.

## AUTHOR CONTRIBUTIONS

*Conceptualization:* Bruce P. Hermann, Joseph I. Tracy, and Aaron F. Struck. *Methodology:* Bruce P. Hermann, Joseph I. Tracy, Aaron F. Struck, and Qirui Zhang. *Visualization:* Aaron F. Struck, Qirui Zhang, Joseph I. Tracy, and Sam S. Javidi. *Supervision:* Bruce P. Hermann and Joseph I. Tracy. *Writing—original draft:* Bruce P. Hermann and Joseph I. Tracy. *Writing—review and editing:* Bruce P. Hermann, Joseph I. Tracy, Aaron F. Struck, and Sam S. Javidi.

## FUNDING INFORMATION

This study was supported by National Institutes of Health/National Institute of Neurological Disorders and Stroke grant R01 NS112816‐01 (J.I.T.).

## CONFLICT OF INTEREST STATEMENT

None of the authors has any conflict of interest to disclose. We confirm that we have read the Journal's position on issues involved in ethical publication and affirm that this report is consistent with those guidelines.

## ETHICS STATEMENT

The authors assert that all procedures contributing to this work comply with the ethical standards of the relevant national and institutional committees on human experimentation and with the Helsinki Declaration of 1975, as revised in 2013. All procedures involving human subjects/patients were approved by the Thomas Jefferson University Institutional Review Board (IRB Number: 19F.754021113).

## PATIENT CONSENT STATEMENT

All study participants signed written informed consent prior to their inclusion in the study.

## Supporting information


Figure S1.

Figure S2.

Figure S3.

Figure S4.

Figure S5.

Appendix S1.

## Data Availability

The data that support the findings of this study are available upon request. The data are not publicly available due to privacy or ethical restrictions.
