# Supplementary material for: Untangling the tangled relationship between cognitive and psychological comorbidities in epilepsy: Bidirectionality and mediation
Source: Epilepsia. 2025 Jul 31;66(12):4972–82. doi: 10.1111/epi.18589 (PMC12779314; doi:10.1111/epi.18589)

Supplement Fig. S1, Factor analysis of PAI: (**a**) Bar graphs shows loadings for each latent factor, with (**b**) scree plot of factor eigenvalues.

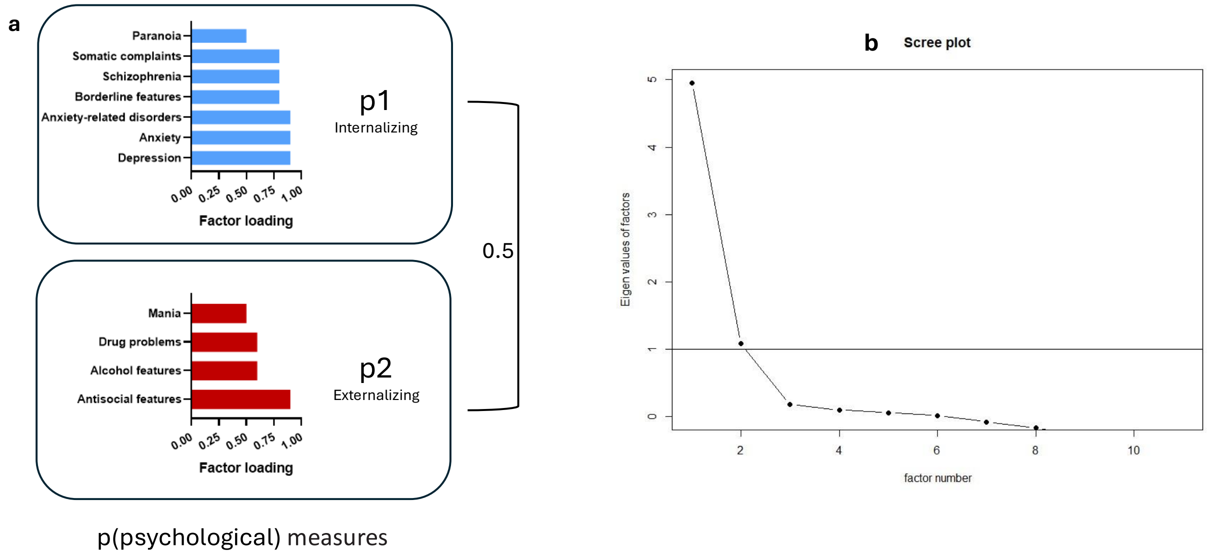

Supplement: Supplementary file 1 — Figure S1. [file EPI-66-4972-s004.pdf]
