# Supplementary material for: Untangling the tangled relationship between cognitive and psychological comorbidities in epilepsy: Bidirectionality and mediation
Source: Epilepsia. 2025 Jul 31;66(12):4972–82. doi: 10.1111/epi.18589 (PMC12779314; doi:10.1111/epi.18589)

Supplement Fig. S2, Factor analysis of cognitive measures: (**a**) Bar graphs shows loadings for each latent factor, with (**b**) scree plot of factor eigenvalues.

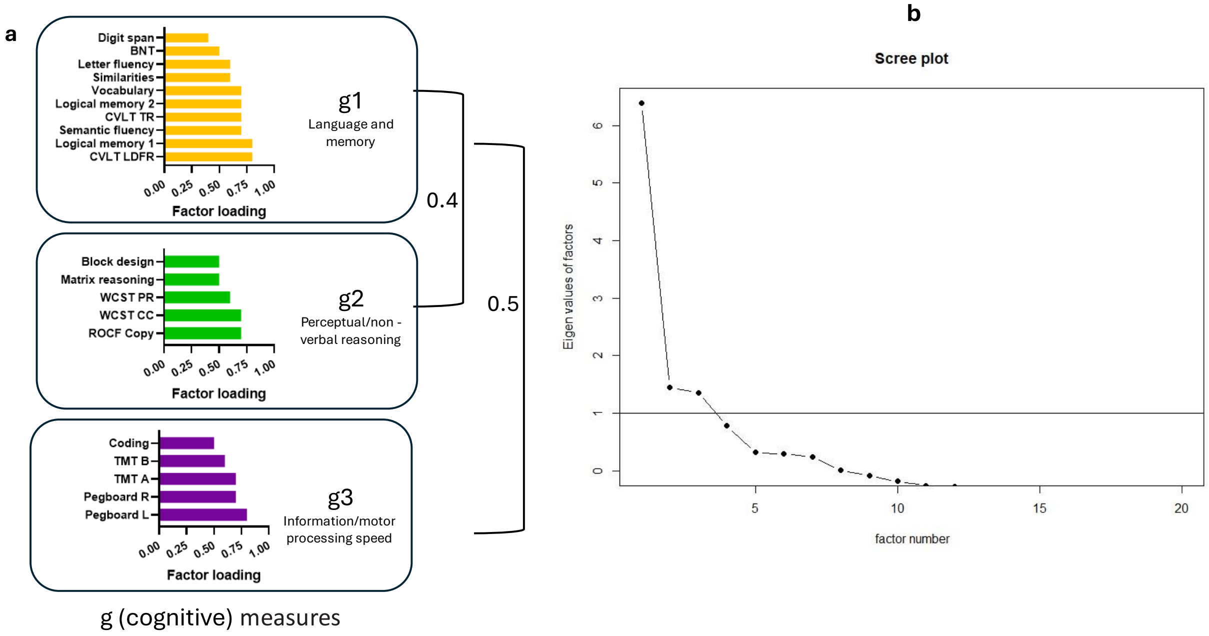

Supplement: Supplementary file 2 — Figure S2. [file EPI-66-4972-s003.pdf]
