# Supplementary material for: Untangling the tangled relationship between cognitive and psychological comorbidities in epilepsy: Bidirectionality and mediation
Source: Epilepsia. 2025 Jul 31;66(12):4972–82. doi: 10.1111/epi.18589 (PMC12779314; doi:10.1111/epi.18589)
Supplement: Supplementary file 3 — Figure S3. [file EPI-66-4972-s006.pdf]

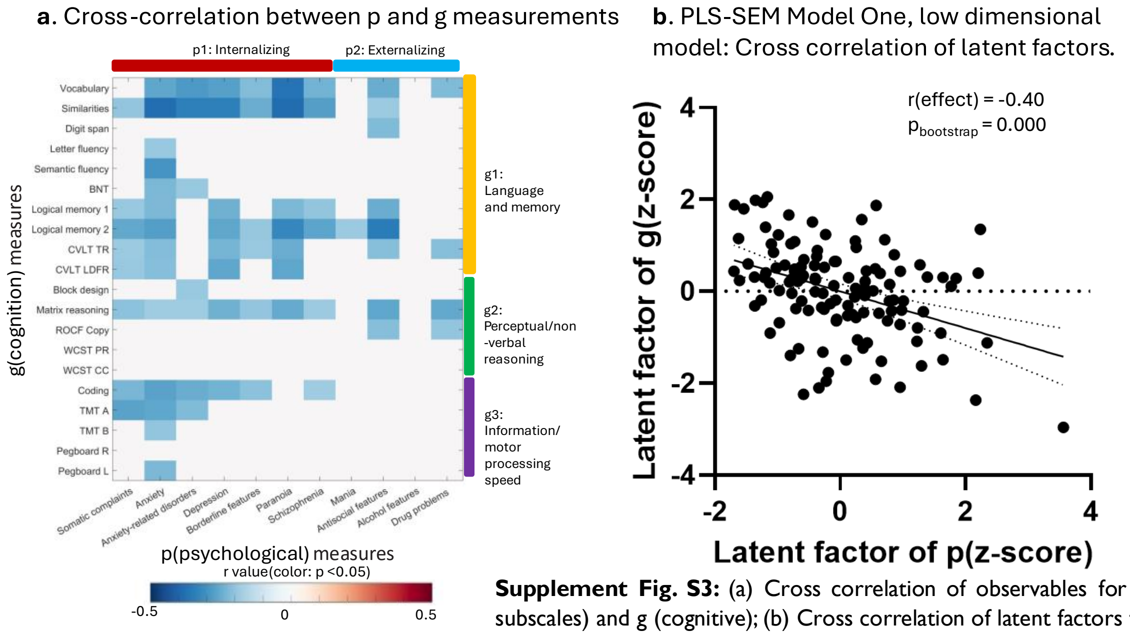

Supplement Fig. S3: (a) Cross correlation of observables for p (PAI subscales) and g (cognitive); (b) Cross correlation of latent factors for low dimensional model (model one).
