# Supplementary material for: Untangling the tangled relationship between cognitive and psychological comorbidities in epilepsy: Bidirectionality and mediation
Source: Epilepsia. 2025 Jul 31;66(12):4972–82. doi: 10.1111/epi.18589 (PMC12779314; doi:10.1111/epi.18589)
Supplement: Supplementary file 4 — Figure S4. [file EPI-66-4972-s002.pdf]

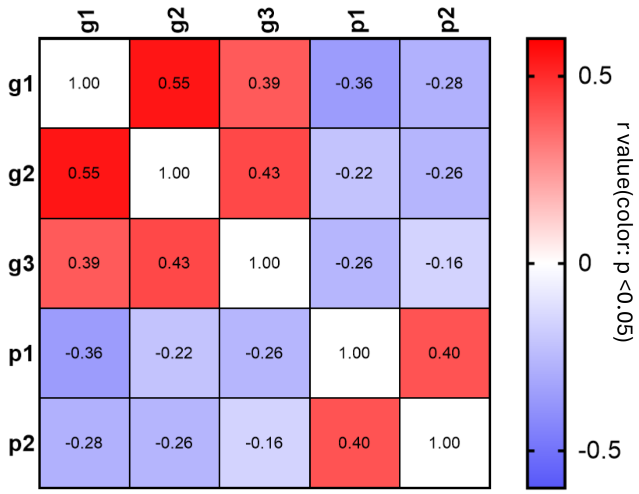

Supplement Fig. S4: Cross correlation of latent factors for high dimensional model (model two). Colorized significance (p values) provided.
