# Supplementary material for: Untangling the tangled relationship between cognitive and psychological comorbidities in epilepsy: Bidirectionality and mediation
Source: Epilepsia. 2025 Jul 31;66(12):4972–82. doi: 10.1111/epi.18589 (PMC12779314; doi:10.1111/epi.18589)
Supplement: Supplementary file 5 — Figure S5. [file EPI-66-4972-s001.pdf]

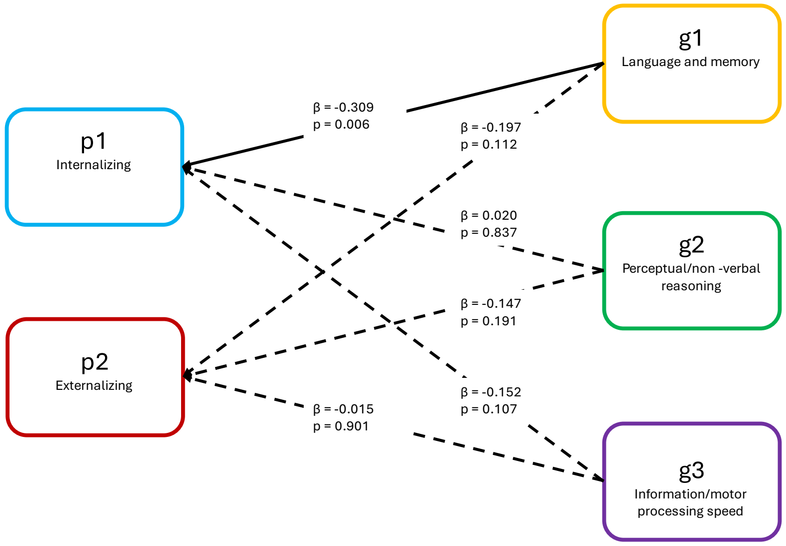

Supplement Fig. S5: PLS-SEM model reversing the direct effects in high dimensional model (model 2): 3 g factors predicting 2 p factors. Path coefficients and p values shown.
