# Supplementary material for: Untangling the tangled relationship between cognitive and psychological comorbidities in epilepsy: Bidirectionality and mediation
Source: Epilepsia. 2025 Jul 31;66(12):4972–82. doi: 10.1111/epi.18589 (PMC12779314; doi:10.1111/epi.18589)
Supplement: Supplementary file 6 — Appendix S1. [file EPI-66-4972-s005.docx]

**Untangling the Tangled Relationship Between Cognitive and Psychological**

**Comorbidities in Epilepsy: Bi-directionality and Mediation**

**Authors: Bruce P. Hermann^1^, Aaron F. Struck^1^, Qirui Zhang^2^, Sam S Javidi^2^, Joseph I Tracy^2^***

**^1^ University of Wisconsin, Madison, WI, USA**

**^2^ Farber Institute for Neuroscience, Department of Neurology, Thomas Jefferson University; Philadelphia, PA, USA**

***Corresponding author. Email:** [**joseph.tracy@jefferson.edu**](mailto:joseph.tracy@jefferson.edu)

**SUPPLEMENTARY MATERIALS**

Participants:

All patients were candidates approved for thermal ablation or resection of the ictal temporal lobe (details provided in Supplementary Materials). The temporal lobe seizure focus was determined by multi-modal data (surface video EEG, MRI, PET, neuropsychological assessment) and a consensus decision of the TJUCEC surgical conference committee (Sperling, 1993; Sperling et al., 1992). Diagnosis was further adjudicated through clinical history, clinical interview, and chart review from all providers. Exclusion criteria consisted of medical illness with central nervous system impact other than epilepsy; prior or current alcohol or illicit drug abuse; evidence of extra-temporal seizures or MRI/PET pathology; and contraindications to MRI; psychiatric hospitalization for any Axis I disorder listed in Diagnostic and Statistical Manual of Mental Disorders, Fifth Edition. Because depression displays a high comorbidity with epilepsy, depressive disorders were allowed (Kanner et al., 2024). As noted, the TLE sample demographics, clinical characteristics, and mean values on the PAI subtests and cognitive measures are provided in manuscript Table 1.

Factor Analysis*:* Sample measure adequacy for each test and an overall KMO factor greater than 0.8 is consider meritorious (Kaiser & Rice, 1974). Individual measures of sampling adequacy were also collected for each item. Any individual tests with KMO factor <0.6 were excluded from the factorization. Bartlett correlation tests were performed to determine whether the correlations were sufficient for factor analysis. To determine the optimal number of factors, the minimum average partial test was performed looking at both the 1976 (Velicer, 1976) and 2000 criteria (Velicer et al., 2000) and scree plots. Gradient projection with oblique rotations were used for factor rotation. Analysis was performed in R (R Foundation version 4.4 Vienna Austria) using the “GPA rotation ” (Bernaards & Jennrich, 2005) and “EFA dimensions” (O’Connor, 2024) packages.

Partial Least Squares Structural Equation Models: All models included bootstrapping to increase statistical power, noting that bootstrapping makes no assumptions about the shape of each variables distribution or the sampling distribution of the test statistic, and can be applied to small sample sizes (Hair et al., 2017) . Lastly, ensuring that collinearity was not at critical levels and that the path coefficients are not biased, we report that the variance inflation factor of each indicator was below acceptable levels (<5; (Hair et al., 2017). A significant direct effect (permutation Bonferroni [bonf]-p < 0.05) indicated a reliable dependency of the cognitive LF’s on the psychological LF’s with a positive value indicating that a unit change in the independent variable (psychological LF) was associated with a unit change in the dependent variable (the cognitive LF). In our mediation models, we re-constructed our high and low PLS-SEM models with a measure of functional reserve inserted as a mediator of the relationship between our “p” and “g” LF’s. Indirect effect coefficients quantified a separate potential causal chain(s) from “p” to “g” dimensions. Each PLS-SEM model was run with years of education modelled as a weighted (covariate) vector, yielding model coefficients that accounted for this factor. Note, partial least squares regression is the preferred form of regression to use if there are correlations among the independent variables and the number of predictors is high relative to the sample size.

**SUPPLEMENTARY TABLES AND FIGURES**

**Supplement Table 1:** Personality Assessment Inventory (PAI) scales and indices (Morey, 1991).

**Supplement Table 2.** Relevant statistics from the factor analysis of the PAI and the cognitive measures.

**Supplement Table S3.** Five-fold cross validation of the 2-factor model for our (p) psychological measures and the 3-factor model for our (g) cognitive measures.

**Supplement Figure 1,** Factor analysis of PAI: (**a**) Bar graphs show loadings for each latent factor, with (**b**) scree plot of factor eigenvalues.

**Supplement Figure 2,** Factor analysis of cognitive measures: (**a**) Bar graphs show loadings for each latent factor, with (**b**) scree plot of factor eigenvalues.

**Supplement Figure 3:** (a) Cross correlation of observables for p (PAI subscales) and g (cognitive); (b) Cross correlation of latent factors for low dimensional model (model one).

**Supplement Figure 4:** Cross correlation of latent factors for high dimensional model (model two). Colorized significance (p values) provided.

**Supplement Figure 5:** PLS-SEM model reversing the direct effects in high dimensional model (model 2): 3 g factors predicting 2 p factors. Path coefficients and p values shown.

Table S1. Personality Assessment Inventory (PAI) scales and indices (Morey, 1991).

| **Clinical scales** | **Description** |
| --- | --- |
| Somatic Complaints (SOM) | Physical concerns and complaints |
| Anxiety (ANX) | General feelings of tension, worry, and nervousness |
| Anxiety-related Disorders (ARD) | Specific anxiety symptoms that relate to different categories of anxiety disorders |
| Depression (DEP) | General feelings of worthlessness, sadness, and lethargy |
| Mania (MAN) | Level of high energy and excitability |
| Paranoia (PAR) | Suspiciousness and concern about others harming them |
| Schizophrenia (SCZ) | Unusual sensory experiences, bizarre thoughts, and social detachment |
| Borderline Features (BOR) | Problems with identity, emotional instability, and problems with friendships |
| Antisocial Features (ANT) | Level of cruel/criminal behavior and selfishness |
| Alcohol Features (ALC) | Problems with excessive drinking |
| Drug Problems (DRG) | Problems with excessive recreational drug use |

Table S2 Relevant statistics for the factor analysis of the PAI and the cognitive measures.

|  | KMO | Bartlett Correlation Test | RMSEA index | Multiple R-Square for scores with factors | | |
| --- | --- | --- | --- | --- | --- | --- |
| Cognitive | 0.80 | P<0.001 | 0.14 | F1: 0.91 | F2: 0.84 | F3: 0.84 |
| PAI | 0.87 | P<0.001 | 0.1 | F1: 0.94 | F2: 0.85 |  |

**Table S3.** *Five-fold cross validation of the 2-factor model for our (p) psychological measures and the 3-factor model for our (g) cognitive measures.*

Psychological Variables (p): Two factor model


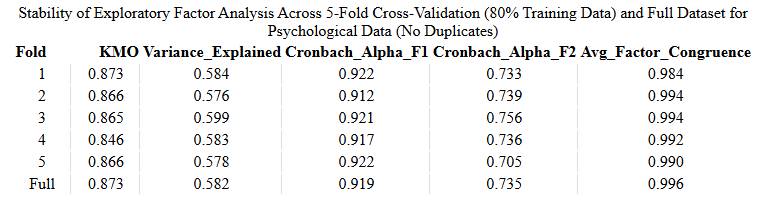


Cognitive Variables (g): Three factor Model


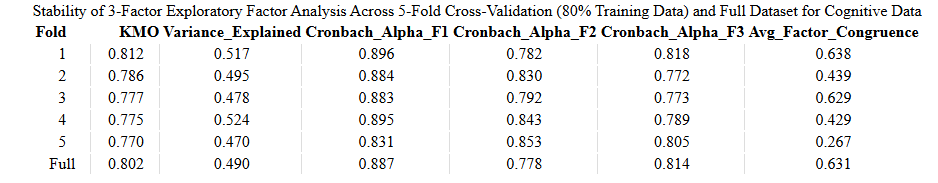


As can be seen for both cross-validations the Kaiser-Meyer-Olin (KMO) Test values all indicate the data is suitable for factor analysis. KMO is calculated based upon the correlation between the variables. KMO is a measure of the proportion of variance among the variables that might be common variance. Generally, KMO values between .5 and 1.0 are considered acceptable. Note, for both the psychological and psychological data the KMO values across the validations are within that range.

The levels of total variance explained across the validations is slightly higher for the psychological measures (Full=.582; cognitive measures, Full=.490), but both are at substantive levels. This measure of total variance captures the cumulative variance of the two factors in the (p) psychological model or the three factors in the (g) cognitive model.

The Cronbach’s Alpha values in the table below represents the internal consistency of each factor, indicating that variables within the factor are measuring the same underlying construct. A value of .70 or higher is acceptable. For the psychological (p) model the values exceed .91 for all folds. For the cognitive (g) model the values exceed .83 for all folds.

**REFERENCES**

Bernaards, C. A., & Jennrich, R. I. (2005). Gradient Projection Algorithms and Software for Arbitrary Rotation Criteria in Factor Analysis. *Educational and Psychological Measurement*, *65*(5), 676-696. https://doi.org/10.1177/0013164404272507

Hair, J. F., Hult, G. T. M., Ringle, C. M., & Sarstedt, M. (2017). *A Primer on Partial Least Squares Structural Equation Modeling*. Sage Publishers.

Kaiser, H. F., & Rice, J. (1974). Little jiffy, mark IV. *Educational and Psychological Measurement*, *34*(1), 111-117.

Kanner, A. M., Shankar, R., Margraf, N. G., Schmitz, B., Ben-Menachem, E., & Sander, J. W. (2024). Mood disorders in adults with epilepsy: a review of unrecognized facts and common misconceptions. *Ann Gen Psychiatry*, *23*(1), 11. https://doi.org/10.1186/s12991-024-00493-2

O’Connor, B. (2024). *R package 'EFA.dimensions' - Exploratory Factor Analysis Functions for Assessing Dimensionality*.

Sperling, M. R. (1993). Neuroimaging in epilepsy: recent developments in MR imaging, positron-emission tomography, and single-photon emission tomography. *Neurol Clin*, *11*(4), 883-903.

Sperling, M. R., O'Connor, M. J., Saykin, A. J., Phillips, C. A., Morrell, M. J., Bridgman, P. A., French, J. A., & Gonatas, N. (1992). A noninvasive protocol for anterior temporal lobectomy. *Neurology*, *42*(2), 416-422. https://doi.org/10.1212/wnl.42.2.416

Velicer, W. F. (1976). Determining the number of components from the matrix of partial correlations. *Psychometrika*, *41*(3), 321-327.

Velicer, W. F., Eaton, C. A., & Fava, J. L. (2000). Construct explication through factor or component analysis: A review and evaluation of alternative procedures for determining the number of factors or components. *Problems and solutions in human assessment: Honoring Douglas N. Jackson at seventy*, 41-71.
